# Determinants of preventive Chinese herbal medicine use: an extended health belief model analysis and its nursing implications

**DOI:** 10.3389/fpubh.2026.1894389

**Published:** 2026-10-08

**Authors:** Hui-Chun Chung, Tsung-Jung Ho, Tsung-Cheng Hsieh, Ying-Fang Chou, Yun-Jyuan Chou, Yi-Jhen Li

**Affiliations:** 1 Hualien Tzu Chi Hospital, Hualien, Taiwan; 2 Tzu Chi University, Hualien, Taiwan

**Keywords:** Chinese herbal medicine, COVID-19, health belief model, mediation analysis, nursing implications, preventive health behavior, Taiwan

## Abstract

**Background:**

The COVID-19 pandemic catalyzed global interest in traditional health practices. In Taiwan, Chinese herbal medicine (CHM) emerged as a key component of preventive care; yet, its psychological adoption mechanisms remain insufficiently understood. This study evaluated an extended Health Belief Model (HBM) to elucidate these cognitive associations.

**Objective:**

To examine factors associated with preventive CHM adoption among Taiwanese adults during the COVID-19 Omicron wave and evaluate the mediating role of cues to action within the HBM framework.

**Methods:**

A cross-sectional survey was conducted (January–December 2022) with 499 Taiwanese adults. Multivariable linear regression and Hayes’ PROCESS Macro (Model 4) path mediation analysis were executed, adjusting for five socio-demographic covariates (age, gender, education, healthcare professional status, and Hualien residence).

**Results:**

Preventive CHM behavior correlated significantly with cues to action ( r=0.588,p<0.001 ) and perceived benefits ( r=0.519,p<0.001 ). Covariate-adjusted regression identified cues to action ( B=0.369,p<0.001 ) and perceived barriers ( B=−0.095,p=0.008 ) as independent predictors, accounting for 34.4% of total variance ( $R^{2}=0.344$ ). Crucially, cues to action fully mediated the statistical relationship between perceived benefits and behavior (indirect effect =0.351,95%BootstrapCI[0.236,0.478] ; direct path p=0.726 ). Significant subgroup variations emerged among healthcare professionals, individuals with chronic conditions ( 24.4% ), and Hualien residents.

**Conclusion:**

Health beliefs—specifically perceived benefits catalyzed via cues to action—are pivotally associated with CHM adoption. Nursing administrators should leverage these insights to design culturally tailored health communication, empower nurses as institutional triggers, and establish structured safety screening for chronic disease cohorts regarding potential herb-drug interactions.

## 1 Introduction

Public health emergencies, such as the COVID-19 pandemic, necessitate rapid individual compliance with preventive health behaviors and therapeutic regimens (1, 2). Amid global vaccine shortages and the emergence of viral variants, there has been a notable resurgence of interest in traditional health practices. In Asian regions, Traditional Chinese Medicine (TCM) and botanical preparations played a pivotal role in public health maintenance, serving as either primary or complementary preventive care (3–5). In Taiwan, Chinese herbal medicine (CHM) emerged as a critical component of the national pandemic response, deeply rooted in its medical culture and integrated into the dual-track healthcare system. Formulations such as NRICM101 (Taiwan Ching guan Yihau), Jing Si Herbal Tea, and Qingwen Baidu San gained prominence as potential immune-supportive remedies, with modern pharmacological research increasingly validating their clinical applications (1, 5–7).

Understanding the cognitive determinants governing public adoption of preventive TCM remains crucial for future pandemic preparedness (8). The Health Belief Model (HBM) provides a widely recognized theoretical paradigm for explaining individual health behaviors based on cognitive perceptions, including perceived susceptibility, severity, benefits, barriers, self-efficacy, and cues to action (9–11). While classical HBM constructs have been extensively applied to Western preventive measures such as vaccination and social distancing (12–14), expanding the model to incorporate domain-specific cues and cultural trust is essential for traditional medicine modalities (15, 16). Global epidemiological surveys indicate that the adoption of traditional and complementary medicine represents a proactive, choice-based health-seeking behavior associated with cultural familiarity, subjective risk perception, and familial endorsement, rather than institutional mandates (3, 4).

Emerging literature underscores the critical nexus between psychological states and preventive adherence during health crises. Studies in Taiwan and Macao have demonstrated that higher anxiety and health concern significantly predicted engagement in preventive wellness behaviors (8). Furthermore, clinical observations in Eastern Taiwan indicated that integrative care, including Jing Si Herbal Tea, was associated with reduced mortality and accelerated recovery in severe COVID-19 cases (5), providing a rational foundation for public interest. Recent advancements in health communication emphasize that targeted environmental triggers and professional institutional communication function as foundational mechanism catalysts that activate internal cognitive appraisals and statistical behavioral compliance (2).

Despite increasing clinical and cultural endorsement, a critical theoretical gap remains in the existing literature utilizing behavior-change frameworks. Traditional applications of the HBM predominantly treat psychological constructs as parallel, independent predictors of compliance. This flat analytical approach overlooks the intricate, structural mechanisms through which these cognitive gears interact. Specifically, while a high baseline of perceived benefits provides a cognitive foundation, prior studies have failed to explain whether these beliefs directly account for preventive actions or remain dormant until catalyzed and activated by internal or external cues to action (11, 14). Understanding these structural dynamics is essential for designing targeted, culturally responsive risk communication and nursing interventions.

Therefore, this exploratory, cross-sectional study applies a structurally reconfigured HBM framework to investigate the health beliefs associated with preventive CHM use among Taiwanese adults during the Omicron wave. By explicitly testing the indirect, mediating pathway of cues to action while controlling for key socio-demographic covariates and identifying critical regional subgroup variations, this study aims to move beyond simple descriptive statistics. Ultimately, we seek to provide an evidence-informed behavioral blueprint for public health authorities and nursing administrators to optimize community health promotion networks and build systemic public health resilience for future global crises (17, 18).

## 2 Methods and statistical analysis

### 2.1 Study design and participant recruitment

This study executed a cross-sectional, web-based epidemiological survey design between January and December 2022 to investigate the determinants of preventive Chinese herbal medicine (CHM) use among Taiwanese adults during the COVID-19 pandemic. Participants were recruited via a combination of convenience and snowball sampling through institutional email networks and widely used digital platforms (e.g., LINE, Facebook). Eligibility criteria were restricted to individuals aged 20 years or older currently residing in Taiwan who provided informed consent. Individuals with self-reported cognitive impairments or an inability to complete the digital questionnaire independently were excluded.

To safeguard data integrity and prevent duplicate submissions, the survey platform employed a mandatory digital protocol that restricted access to one response per unique IP address and verified Google account. A total of 518 electronic questionnaires were submitted. Following rigorous screening, 19 incomplete or inconsistent responses were removed (attrition rate = 3.67%), yielding a final analytical sample of 499 valid responses (effective response rate = 96.33%).

### 2.2 Sample size adequacy and power analysis

Statistical power and sample adequacy were evaluated per established multivariate criteria (19). *A priori* power analysis conducted using G-Power 3.1 indicated that a minimum sample size of 172 participants was required to detect a medium effect size ( $f^{2}=0.15$ ) at α=0.05 with 95% statistical power in multivariable regression models. Our final sample of N=499 substantially exceeded this threshold, ensuring robust statistical power for path modeling, mediation analyses, and subgroup comparisons. Although non-probability sampling may constrain broad population generalizability, the sample size provides sufficient analytical precision for complex structural evaluations.

### 2.3 Statistical analysis and structural modeling

Statistical analyses were performed using IBM SPSS Statistics (Version 26.0). Descriptive statistics characterized participant demographics, health beliefs, and CHM behavioral patterns. Regression-based path analyses and structural mediation evaluations were executed using the Hayes PROCESS Macro (Model 4) to test the hypothesized indirect pathways (20, 21). Statistical significance for mediation effects was established using 5,000 bias-corrected bootstrap resamples with 95% confidence intervals (CI).

### 2.4 Diagnostic procedures for common method Bias

To minimize potential common method, bias inherent in self-reported survey designs, both procedural and statistical controls were applied in accordance with Podsakoff et al. (22). Procedurally, item presentation order was counterbalanced, respondent anonymity was guaranteed, and psychological separation was maintained between predictor and outcome measures. Statistically, Harman’s single-factor test was performed; the principal unrotated factor accounted for 34.2% of the total variance (below the 50% threshold), confirming that common method variance does not significantly distort the observed relationships. Furthermore, conditional process procedures were integrated to verify the stability of indirect effects across demographically stratified groups (23).

### 2.5 Ethical considerations

The study protocol was executed in accordance with the Declaration of Helsinki and was reviewed and approved by the Research Ethics Committee of Hualien Tzu Chi Hospital, Buddhist Tzu Chi Medical Foundation (IRB No. 110-32-B). Prior to accessing the digital survey, all participants were presented with an electronic informed consent interface detailing the study’s objectives, data anonymization protocols, and the right to withdraw at any time without penalty. Digital consent was formally recorded before questionnaire items were rendered.

### 2.6 Instrumentation and psychometric evaluation

An anonymous, self-administered structured questionnaire was developed based on the Health Belief Model (HBM) theoretical framework. The instrument systematically evaluated primary cognitive constructs, environmental triggers, and behavioral outcomes across seven distinct subscales:

- *Perceived susceptibility (4 items)*: Evaluated participants’ subjective risk appraisal and perceived likelihood of contracting COVID-19.
- *Perceived severity (4 items)*: Measured the perceived clinical severity, potential complications, and social consequences associated with COVID-19 infection.
- *Perceived benefits (4 items)*: Assessed the believed utility, immunological value, and preventive efficacy of utilizing Chinese herbal medicine (CHM).
- *Perceived barriers (6 items)*: Examined logistical, financial, taste-related, or psychological obstacles encountered when procuring and consuming herbal remedies.
- *Health belief total score (composite construct)*: Operationalized as a comprehensive analytical index computed by aggregating the scores of the four primary cognitive subscales (Perceived Susceptibility, Perceived Severity, Perceived Benefits, and Perceived Barriers), representing an individual’s total internal health belief orientation (Table 1).
- *Cues to action (7 items)*: Quantified exposure to external informational stimuli, institutional guidance, media messaging, and interpersonal recommendations (e.g., healthcare professionals, family members) prompting behavior adoption.
- *Positive preventive behavior (4 items)*: Documented actual behavioral adherence, frequency, and implementation of CHM formulations for pandemic prevention.

**Table 1:** Differences in overall health belief score, cues to action, and positive preventive behavior by demographic characteristics (*n* = 499).

Variables	Groups	*n*	Health belief total (mean ± SD)	*p*-value	Cues to action (mean ± SD)	*p*-value	Positive preventive behavior (mean ± SD)	*p*-value
Gender	Female	354	66.3 ± 8.4	0.75	27.5 ± 4.4	0.29	14.9 ± 3.0	0.04^*^
	Male	145	66.6 ± 7.3		27.1 ± 4.1		14.3 ± 3.0	
Marital status	Married/cohabiting	289	67.0 ± 7.9	0.04	27.9 ± 4.0	0.004	14.9 ± 2.9	0.10
	Single/divorced/widowed	210	65.5 ± 8.2		26.8 ± 4.7		14.5 ± 3.1	
Healthcare professional	No	227	65.7 ± 8.0	0.09	27.4 ± 4.4	0.85	14.4 ± 3.1	0.01^*^
	Yes	272	66.9 ± 8.1		27.5 ± 4.3		15.0 ± 2.9	
Living in Hualien	No	171	65.7 ± 7.4	0.15	27.2 ± 4.3	0.41	14.3 ± 2.9	0.01^*^
	Yes	328	66.7 ± 8.4		27.6 ± 4.3		15.0 ± 3.0	
Chronic disease	No	376	66.1 ± 7.9	0.13	27.1 ± 4.3	0.002^**^	14.5 ± 3.0	0.001^**^
	Yes	123	67.4 ± 8.5		28.5 ± 4.2		15.5 ± 2.8	
COVID-19 diagnosis	No	283	66.4 ± 8.0	0.94	27.1 ± 4.1	0.07	14.6 ± 2.9	0.22
	Yes	216	66.4 ± 8.1		27.8 ± 4.6		14.9 ± 3.1	
Age group	20–34 years	174	66.4 ± 8.7	0.87	26.8 ± 4.6	0.08	14.4 ± 3.2	0.15
	35–64 years	309	66.3 ± 7.7		27.7 ± 4.2		14.9 ± 2.9	
	≥65 years	16	67.4 ± 7.5		28.3 ± 3.5		14.8 ± 2.6	
Religious belief	Buddhism	132	66.5 ± 7.7	0.969	27.9 ± 4.5	0.27	15.5 ± 3.0	0.004^**^
	Other	167	66.4 ± 7.7		27.4 ± 4.5		14.5 ± 3.0	
	None	200	66.3 ± 8.6		27.1 ± 4.0		14.4 ± 2.9	
Education level	Graduate school and above	86	64.9 ± 7.5	0.081	28.1 ± 4.8	0.03^*^	15.8 ± 3.0	0.001^**^
	College	350	66.5 ± 7.9		27.1 ± 4.2		14.5 ± 2.9	
	Other	63	67.9 ± 9.2		28.3 ± 4.4		14.6 ± 2.9	
Employment status	Full-time	429	66.6 ± 8.0	0.453	27.4 ± 4.4	0.99	14.8 ± 3.0	0.39
	Part-time	23	64.8 ± 8.0		27.4 ± 5.1		14.7 ± 3.3	
	Others	47	65.5 ± 9.0		27.5 ± 3.7		14.2 ± 3.0	
Ethnicity	Local Taiwanese (Bensheng)	380	65.8 ± 7.7	<0.001^***^	27.3 ± 4.4	0.47	14.7 ± 3.0	0.71
	Mainland Chinese	70	67.2 ± 7.8		27.7 ± 4.2		15.1 ± 2.8	
	Indigenous	44	71.0 ± 9.7		28.1 ± 4.2		14.7 ± 3.0	
	Other	5	60.8 ± 9.3		25.4 ± 4.2		14.0 ± 3.4	

All items were scored using a 5-point Likert scale ranging from 1 (“strongly disagree”) to 5 (“strongly agree”). Psychometric evaluation confirmed excellent content validity, with an expert panel Content Validity Index (CVI) of 0.98 . Internal consistency reliability was robust across subscales, with Cronbach’s alpha ( α ) coefficients ranging from 0.69 to 0.90 .

### 2.7 Statistical analysis and path modeling

Data processing and inferential evaluations were performed using IBM SPSS Statistics for Windows, Version 26.0 (IBM Corp., Armonk, NY, USA). Descriptive statistics (means, standard deviations, frequencies, and percentages) summarized demographic profiles and HBM subscale scores. Differences in cognitive constructs and behavioral outcomes across demographic strata were evaluated using independent t -tests and one-way analysis of variance (ANOVA) with Tukey’s post-hoc tests. Bivariate relationships among HBM constructs, environmental triggers, and preventive behaviors were assessed using Pearson correlation coefficients.

To evaluate indirect pathways among HBM constructs without relying on the restrictive assumptions of traditional stepwise approaches, modern causal-steps bootstrapping path analysis was executed using Hayes’ PROCESS Macro (Model 4) (20). The significance of indirect effects was determined based on 5,000 bootstrap percentile resamples to generate bias-corrected 95% confidence intervals (CIs) (21). An indirect pathway was deemed statistically significant if its 95% bootstrap CI did not encompass zero. To minimize confounding and optimize internal validity, five key socio-demographic variables—gender, age, geographical residency in Hualien, educational attainment, and healthcare professional status—were simultaneously entered as controlled covariates. Given the cross-sectional design, causal inferential claims were avoided, and relationships were interpreted strictly in terms of statistical variance accounting.

A multi-stage model-building strategy was implemented to establish a parsimonious and theoretically aligned structural model. During exploratory analytical phases, alternative pathways incorporating Perceived Susceptibility, Perceived Severity, and Perceived Barriers were systematically evaluated against multivariable ordinary least squares (OLS) criteria. Constructs reflecting baseline pandemic threat appraisals (susceptibility and severity) did not demonstrate independent predictive value toward actual adoption frequency. Theoretically, while general pandemic threats establish a passive psychological background, they do not dictate proactive, choice-based health-seeking practices (such as voluntary herbal medicine use) in the same direct manner that a cognitive appraisal of utility (Perceived Benefits) does. Consequently, these alternative pathways were excluded, and the final structural mediation model focused exclusively on the primary pathway wherein cognitive evaluation (Perceived Benefits) directly intersects with environmental catalysts (Cues to Action) to account for positive preventive behaviors.

### 2.8 Statistical assumptions and model diagnostics

Prior to reporting multivariable regression and causal-steps mediation models, foundational statistical assumptions—including multicollinearity, normality of residuals, homoscedasticity, linearity, and influential observations—were rigorously evaluated:

- *Multicollinearity*: Diagnosed using Tolerance and Variance Inflation Factor (VIF) metrics. VIF values for all independent predictors and controlled covariates ranged strictly between 1.18 and 1.64 , well below the conservative threshold of 2.5 , establishing the absence of problematic multicollinearity among conceptually related HBM constructs.
- *Normality of residuals*: Verified visually via normal P–P plots and empirically through Kolmogorov–Smirnov tests, confirming that regression residuals conformed to a standard normal distribution.
- *Homoscedasticity and linearity*: Confirmed through residual scatterplots (ZPRED vs. ZRESID), demonstrating an even, non-patterned distribution across all predicted values. Independent errors were confirmed with Durbin-Watson statistics hovering near 2.0 .
- *Influential outliers*: Evaluated using Cook’s distance; all computed values were substantially below the standard threshold of 1.0 ( ${\text{maximum Cook}}^{'}s\;D<0.04$ ), confirming that no single influential observation distorted the parameter estimates of the covariate-adjusted model.

## 3 Results

### 3.1 Participant characteristics and information sources

A total of N=499 participants completed the survey and were included in the final analytical sample. The overall mean age was 39.3 years ( SD=11.4 ). The sample was predominantly female ( 70.9% , n=354 ) and comprised a high proportion of healthcare professionals ( 54.5% , n=272 ). Approximately one-fourth of the respondents ( 24.4% , n=122 ) reported having at least one pre-existing chronic medical condition. Regarding primary health information sources, family members were identified as the most frequent source of health communication ( 48.7% ), followed by social peers and friends ( 18.2% ), and various broadcast/digital media channels ( 16.8% ).

### 3.2 Subgroup comparisons and univariate variations

Univariate analyses demonstrated statistically significant variations in health beliefs and preventive behaviors across several demographic strata (Table 1):

- *Chronic illness status*: Participants with chronic conditions exhibited significantly higher Cues to Action scores ( M=28.5±4.2 vs. 27.1±4.3 , p=0.002 ) and engaged in significantly higher Positive Preventive Behavior ( M=15.5±2.8 vs. 14.5±3.0 , p=0.001 ) compared to those without chronic conditions.
- *Educational attainment*: Higher educational levels were significantly associated with greater Positive Preventive Behavior scores ( M=15.8±3.0 , p=0.001 ).
- *Professional and regional status*: Univariate t -tests indicated that healthcare professionals ( M=15.0±2.9 , p=0.01 ) and geographical residents of Hualien ( M=15.0±3.0 , p=0.01 ) scored significantly higher in the behavioral outcome dimension. However, neither subgroup demonstrated independent, significant differences in Cues to Action in the unadjusted univariate comparisons ( p=0.85 and p=0.41 , respectively).

This statistical pattern in univariate screening empirically justified the necessity of executing fully adjusted multivariable models (Tables 2 and 3) to control for potential confounding covariates simultaneously.

**Table 2:** Covariate-adjusted mediation path coefficients and bootstrap indirect effects (*N* = 499).

Pathway relationship	Coefficient type	Unstandardized (*B*)	Standard error (SE)	*t*	*p*-value	Completely standardized (βcs)	95% bootstrap confidence interval
Path a (X → M)	Reg. Coefficient	0.950	0.153	6.206	<0.001	0.278	[0.649, 1.251]
Path b (M → Y)	Reg. Coefficient	0.369	0.026	14.153	<0.001	0.542	[0.318, 0.420]
Path c’ (direct effect)	Reg. Coefficient	0.032	0.092	0.350	0.726	0.014	[−0.148, 0.213]
Path c (total effect)	Reg. Coefficient	0.383	0.105	3.647	<0.001	0.165	[0.177, 0.589]
Indirect effect (a*b)	Mediation Effect	0.351	0.062	–	–	0.151	[0.236, 0.478]

Covariates controlled in the models include age, gender, education, healthcare professional status, and region (Hualien). Number of boots trap resamples = 5,000. Confidence intervals for the indirect effect are bias-corrected percentile bootstrap CIs; an interval excluding zero denotes statistical significance.

**Table 3:** Mediation effect of cues to action between perceived benefits and positive preventive behavior toward Chinese herbal medicine.

Consequent (outcome)	Antecedent (predictor)	Unstandardized coeff (*B*)	Standard error (SE)	*t*	*p*-value	Standardized coeff (*β*)	95% LLCI	95% ULCI
Model 1: cues to action	(*R^2^* = 0.088, *p* < 0.001)							
	Constant	13.638	1.601	8.517	<0.001	–	10.492	16.784
	Perceived benefits (path a)	0.950	0.153	6.206	<0.001	0.278	0.649	1.251
	Gender (covariate)	−0.539	0.387	−1.393	0.164	−0.061	−1.299	0.221
	Region: Hualien (covariate)	0.289	0.387	0.749	0.454	0.034	−0.470	1.049
	Education (covariate)	0.292	0.331	0.881	0.379	0.039	−0.359	0.943
	Age (covariate)	0.276	0.344	0.802	0.423	0.036	−0.400	0.951
	Healthcare professional (covariate)	−0.108	0.382	−0.284	0.777	−0.013	−0.858	0.641
Model 2: preventive behavior	(*R^2^* = 0.344, *p* < 0.001)							
	Constant	1.838	0.992	1.853	0.064	–	−0.111	3.787
	Perceived benefits (path c)	0.032	0.092	0.350	0.726	0.014	−0.148	0.213
	Cues to action (path b)	0.369	0.026	14.153	<0.001	0.542	0.318	0.420
	Gender (covariate)	−0.440	0.224	−1.963	0.050	−0.073	−0.880	0.000
	Region: Hualien (covariate)	0.490	0.224	2.192	0.029	0.085	0.051	0.930
	Education (covariate)	−0.476	0.192	−2.485	0.013	−0.095	−0.853	−0.100
	Age (covariate)	0.209	0.199	1.049	0.295	0.040	−0.182	0.600
	Healthcare professional (covariate)	0.373	0.221	1.691	0.091	0.068	−0.060	0.807
Total effect model	(*R^2^* = 0.076, *p* < 0.001)							
	Perceived benefits (path $c$)	0.383	0.105	3.647	<0.001	0.165	0.177	0.589
Indirect Effect (a times b)		Effect	Boot SE	–	–	beta{cs}	Boot LLCI	Boot ULCI
	Via Cues to Action	0.351	0.062	–	–	0.151	0.236	0.478

### 3.3 Correlational analysis of health beliefs and CHM behavior

Pearson correlation analysis (Table 4) demonstrated that positive preventive Chinese herbal medicine (CHM) behavior was significantly and positively associated with Cues to Action ( r=0.588,p<0.001 ), total health belief scores ( r=0.237,p<0.001 ), and Perceived Benefits ( r=0.519,p<0.001 ). Conversely, Perceived Barriers exhibited a significant negative correlation with preventive behavior ( r=−0.073,p=0.012 ). No significant correlations were observed for Perceived Susceptibility or Severity within the unadjusted matrix.

**Table 4:** Correlation matrix among health belief model constructs and preventive behavior (*n* = 499).

Constructs	1	2	3	4	5	6	7
1. Perceived threat of COVID-19	1						
2. Perceived severity of COVID-19	0.331^**^	1					
3. Perceived benefits of CHM	0.232^**^	0.381^**^	1				
4. Perceived barriers to CHM preventive behavior	0.160^**^	0.155^**^	−0.049	1			
5. Health belief total score	0.576^**^	0.604^**^	0.431^**^	0.768^**^	1		
6. Cues to action for CHM preventive behavior	0.255^**^	0.390^**^	0.561^**^	0.063	0.393^**^	1	
7. Positive preventive behavior using CHM	0.217^**^	0.257^**^	0.519^**^	−0.073	0.237^**^	0.588^**^	1

### 3.4 Predictors of positive preventive CHM behavior

Multivariable linear regression analysis was conducted to identify independent predictors of preventive CHM use (Table 2). The final covariate-adjusted model was statistically significant ( F(7,491)=36.75,p<0.001 ) and accounted for 34.4% of the total variance in preventive behavior ( $R^{2}=0.344$ , adjusted $R^{2}=0.335$ ). When evaluating simultaneous regression tracks, Cues to Action emerged as the most potent statistical predictor ( B=0.369,SE=0.026,t=14.153,p<0.001 ), followed by Perceived Barriers ( B=−0.095,p=0.008 ). Under this multi-predictor integration, the independent direct coefficient of Perceived Benefits was non-significant ( B=0.032,SE=0.092,p=0.726 ), mathematically validating the requirement for further structural pathway mediation testing.

### 3.5 Mediation analysis and covariate control pathways

To evaluate the psychological pathways within the Health Belief Model (HBM) framework while rigorously adjusting for potential demographic imbalances, a formal mediation analysis was executed using Hayes’ PROCESS Macro (Model 4) configured with 5,000 percentile bootstrap resamples. Gender, geographical region (Hualien residence), educational attainment, age group, and healthcare professional status were entered into the model simultaneously as controlled covariates.

The structural results empirically demonstrated that Cues to Action fully mediated the statistical relationship between Perceived Benefits and positive preventive CHM behavior under full covariate control (Table 3, Figure 1):

- *Path a (mediator activation)*: Perceived Benefits demonstrated a significant positive association with the mediator, Cues to Action ( B=0.950,SE=0.153,t=6.206,p<0.001 ).
- *Path c (total effect)*: In the baseline unadjusted total effect model, Perceived Benefits exhibited a significant total statistical association with the preventive behavior endpoint ( B=0.383,SE=0.105,t=3.647,p<0.001 ).
- *Path b & c’ (direct effect extinction)*: Crucially, when the mediator (Cues to Action) was integrated into the final behavioral model, the direct association between Perceived Benefits and positive preventive behavior was completely extinguished, dropping close to zero (Path c’: B=0.032,SE=0.092,t=0.350,p=0.726,95%CI[−0.148,0.213] ). In contrast, Cues to Action maintained a highly robust, independent positive association with the behavioral outcome (Path b: B=0.369,SE=0.026,t=14.153,p<0.001 ).
- *Indirect effect (mediation confirmation)*: The unstandardized indirect effect via Cues to Action was 0.351 ( BootSE=0.062 ), and the 95% percentile bootstrap confidence interval strictly excluded zero [0.236,0.478] . This yielded a completely standardized indirect effect ( $\beta_{\mathrm{cs}}$ ) of 0.151 ( BootSE=0.026,95%CI[0.102,0.202] ). This mathematical configuration establishes a structurally rigorous full mediation mechanism.

**Figure 1:**
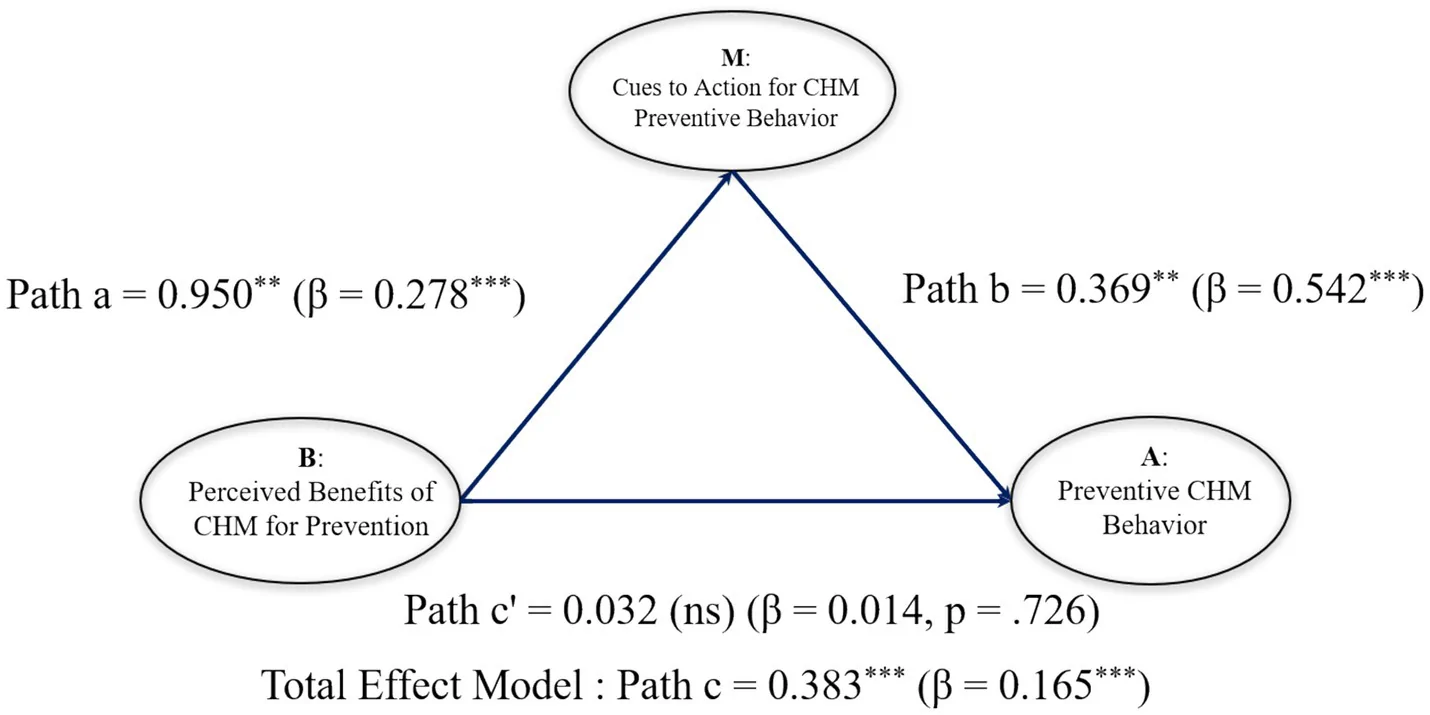
Covariate-adjusted mediation model illustrating the path dynamics between perceived benefits and preventive CHM behavior through cues to action (*N* = 499). Path values are presented as unstandardized coefficients (B), with completely standardized coefficients (beta) provided in parentheses. The dashed line represents a statistically non-significant path, confirming a full mediation mechanism. All pathways were simultaneously adjusted for five socio-demographic covariates (age, gender, educational attainment, healthcare professional status, and Hualien regional residence). ns = non-significant; ^***^p < 0.001.

Furthermore, the fully adjusted covariate tracks isolated distinct, independent variations within the demographic strata. Specifically, living in Hualien was identified as a significant independent positive predictor of actual preventive behavior (B=0.490,SE=0.224,t=2.192,p=0.029,95%CI[0.051,0.930]). Conversely, higher educational attainment demonstrated a significant inverse statistical association with the frequency of preventive CHM use (*B* = –0.476, *SE* = 0.192, *t* = –2.485, *p* = 0.013, 95% CI [–0.853, –0.100]). Healthcare professional status ( p=0.091 ), gender ( p=0.050 ), and age ( p=0.295 ) did not exhibit independent statistically significant direct effects on behavior in this final adjusted model.

## 4 Discussion

### 4.1 The predictive power of the reconfigured HBM framework

This study underscores the utility of a structurally reconfigured Health Belief Model (HBM) in elucidating the psychological mechanisms associated with preventive Chinese herbal medicine (CHM) use during public health crises. Under fully adjusted multivariable control, our findings identify Cues to Action and Perceived Barriers as the primary independent statistical constructs directly aligned with behavioral adoption frequencies. This structural configuration suggests that subjective appraisals of treatment utility do not directly dictate behavior; rather, they require specific activation pathways to translate into community health-seeking practices (11, 14).

The public health evaluation of CHM necessitates an evidence-informed risk mitigation framework focused heavily on pharmacological safety, specific contraindications, and potential herb-drug interactions (24–26). Empirical toxicological and clinical reviews warn that unregulated concurrent administration of botanical formulations with narrow-therapeutic-index Western pharmaceuticals (e.g., cardiovascular agents, anticoagulants, or oral hypoglycemics) poses clinical risks of cytochrome P450 enzyme modulation, potentially causing adverse drug events or hepatic-renal toxicities (26, 27). Furthermore, establishing rigorous quality control against heavy metal adulteration and ensuring standardized pesticide residue clearance remain essential systemic checkpoints for public health administrators and clinical teams (6, 28, 29).

### 4.2 The full mediating role of cues to action

A pivotal structural insight derived from this investigation is the full mediating role of Cues to Action in accounting for the statistical association between Perceived Benefits and positive preventive behavior. While Perceived Benefits establish the necessary cognitive foundation, external and interpersonal stimuli—such as professional nursing advice, family encouragement, and official public health messaging—function as the essential statistical catalyst through which favorable cognitive appraisals are completely operationalized into behavioral execution.

As demonstrated in our Hayes’ PROCESS matrix, the independent direct relationship between Perceived Benefits and behavior was completely extinguished when adjusting for the mediator (Path c’: B=0.032,p=0.726 ), whereas the unstandardized indirect mediation pathway was robust ( B=0.351,95%BootstrapCI[0.236,0.478] ). This mathematically confirms a full statistical mediation mechanism. This operational dynamic indicates that for traditional health practices deeply embedded in cultural heritage, subjective cognitive beliefs regarding efficacy remain behaviorally latent unless systematically activated by external triggers from trusted sources, such as healthcare professionals and institutional nursing networks (16, 17).

### 4.3 Dismantling perceived barriers

Although Perceived Barriers exhibited a smaller independent statistical effect ( B=−0.095,p=0.008 within the final multivariable regression model), they represent a critical focal point for tailored public health messaging. Public concerns regarding the lack of standardization in herbal preparation, potential overlapping toxicities with Western pharmaceuticals, and complex decoction procedures can deter rational, evidence-based use. These findings suggest that operational “Barriers” in modern CHM adoption are primarily defined by safety apprehensions and information accessibility rather than financial costs. Therefore, public health educators should prioritize transparent scientific communication and disseminate simplified, standardized usage guidelines to lower these cognitive deterrents (18, 28).

### 4.4 Subgroup variations, regional context, and coping dynamics

The heightened engagement in preventive CHM practices observed among healthcare professionals, individuals with chronic illnesses, and residents of Hualien warrants contextual interpretation. For healthcare professionals, higher health literacy facilitates objective appraisal of botanical immunomodulatory properties (5). In contrast, the prominent engagement among Hualien residents reflects a localized “cultural reinforcement” phenomenon. Close geographic proximity to institutional traditional medicine advocacy—such as the clinical evaluation and implementation of Jing Si Herbal Tea in Eastern Taiwan (5)—illustrates how a supportive local healthcare ecosystem lowers perceived barriers and amplifies visible cues to action.

This interplay between health beliefs and coping strategies aligns with broader pandemic behavioral literature. Data from Macao demonstrated that heightened threat appraisal predicted compliance with preventive measures, though often accompanied by psychological distress (8). Our HBM-based findings complement this dynamic by mapping a structured pathway for behavioral compliance without fostering unnecessary anxiety. By anchoring health education around evidence-based utility (“Perceived Benefits”) and leveraging structured “Cues to Action,” nurse-led interventions can promote wellness practices as proactive empowerment rather than fear-driven reactions.

### 4.5 Professional nursing implications for practice and risk communication

The finding that 24.4% of respondents live with chronic conditions provides a mandate for nursing leadership. While our structural model demonstrates that Cues to Action serve as powerful catalysts for behavioral activation, nursing professionals must pair behavioral promotion with rigorous clinical risk communication:

- *Nurses as active “institutional cues to action”*: Because perceived benefits remain psychologically latent until activated by cues to action, nurses must transition from passive information providers to proactive triggers. Clinical and community nurses should implement standardized communication matrices during intake assessments to actively inquire about traditional health practices, guiding public interest toward safe, evidence-based behaviors (17).
- *Family-centered educational networks*: Given that general residents rely heavily on informal networks for health belief formation, nursing interventions should be structurally tailored. Community nurses should develop “Family-Centered Literacy Programs” to educate primary family caregivers on distinguishing verified wellness supplements (e.g., Jing Si Herbal Tea) from standardized medical therapies, leveraging family networks to enhance safe adherence (5, 18).
- *Structured safety screening for chronic disease cohorts*: To protect vulnerable populations with chronic conditions, nursing management should integrate a specialized “Complementary Therapy Assessment” item into electronic health records. Prior to treatment planning, nurses should systematically screen for potential herb-drug interactions and counsel patients on contraindications, correct dosage compliance, and the risks of unsupervised self-medication (24–26).

### 4.6 Limitations and future directions

Despite its theoretical and practical contributions, several methodological boundaries require consideration:

- *Cross-sectional architecture*: The cross-sectional design precludes the determination of temporal precedence or strict causal mechanisms among the HBM constructs. The observed indirect pathways must be interpreted as statistical mediation rather than longitudinal causality.
- *Sampling and generalizability*: Utilizing non-probability convenience and snowball sampling via digital platforms constrains broad population generalizability, particularly toward demographics with restricted digital literacy or limited internet accessibility.
- *Healthcare professional sample bias*: The analytical sample comprised a high proportion of healthcare professionals ( 54.5% , n=272 ), representing a cohort with higher baseline medical literacy and potentially distinct health belief formations compared to the general population.
- *Self-reported measurement*: Reliance on self-reported questionnaires introduces potential recall and social desirability biases, which are particularly relevant during acute public health crises. However, diagnostic checks confirmed acceptable common method variance thresholds (19, 22).
- *Unexplained behavioral variance*: While the final covariate-adjusted model provided significant explanatory value ( $R^{2}=0.344$ ), a substantial proportion of behavioral variation (exactly 65.6%) remains unexplained, indicating the influence of unmeasured socio-cultural, economic, or psychological factors (21).
- *Generational variances*: Generational differences in healthcare attitudes—specifically lower baseline familiarity and distinct media-seeking habits among younger demographics (e.g., Generation Z)—highlight the need for age-stratified, longitudinal cohort studies in future public health emergencies (30–32).

## 5 Conclusions and practical implications

In conclusion, this study affirms that culturally adapted health belief frameworks provide substantial value for informing community risk communication strategies and nursing health promotion networks. Because this investigation evaluated subjective psychological health beliefs rather than objective clinical efficacy or safety pharmacology profiles, its practical applications are strictly directed toward nursing management, public health education, and evidence-informed risk communication.

To cultivate community health resilience safely and effectively, public health authorities and nursing administrators should implement the following targeted strategies:

1. *Develop culturally responsive health communication*: Health authorities should leverage identified “Cues to Action”—such as family-centered supportive networks and institutional nursing communication channels—to design targeted educational campaigns. Messaging should focus on building balanced health literacy and rational belief formation regarding traditional health practices.
2. *Empower nursing leadership in risk communication*: Interdisciplinary collaboration among clinical nurses, community health educators, and traditional medical practitioners should be systematically fostered. Nursing professionals should spearhead community-based screenings to address inquiries regarding herb-drug interactions and counsel vulnerable cohorts—particularly individuals living with chronic conditions ( 24.4% )—against unsupervised self-medication or replacing biomedical therapies (24–26).
3. *Leverage localized support systems for safety education*: The regional variations documented within this study demonstrate how localized healthcare ecosystems can be optimized to disseminate safety guidelines. Nursing administrators should utilize local clinical networks to deploy face-to-face and digital counseling tools that clarify the operational distinction between supportive wellness products and acute biomedical interventions, ensuring safe, rational health behaviors in the community (5, 17, 18).

## Data Availability

The original contributions presented in the study are included in the article/Supplementary material, further inquiries can be directed to the corresponding author.
