# Supplementary material for: Determinants of preventive Chinese herbal medicine use: an extended health belief model analysis and its nursing implications
Source: Front Public Health. 2026 Oct 8;14:1894389. doi: 10.3389/fpubh.2026.1894389 (PMC13687336; doi:10.3389/fpubh.2026.1894389)
Supplement: Supplementary file 1 [file Data_Sheet_1.PDF]

Supplementary Table

S1. Botanical Compositions, Formulations, and Clinical Profiles of Key Preventive Chinese Herbal Medicine (CHM) Remedies in Taiwan

Formulation Name	Botanical / Botanical-Mineral Compositions (Latin & Pin-Yin)	Primary Mode of Administration	Reported Mechanism & Preventive Profile
NRICM101<br><br>*(Taiwan Chingguan Yihau)*	*Scutellaria baicalensis* (Huang-Qin), *Houttuynia cordata* (Yu-Xing-Cao), *Morus alba* (Sang-Ye), *Saposhnikovia divaricata* (Fang-Feng), *Trichosanthes kirilowii* (Gua-Lou-Shi), *Isatis indigotica* (Ban-Lan-Gen), *Magnolia officinalis* (Hou-Po), *Mentha canadensis* (Bo-He), *Glycyrrhiza glabra* (Gan-Cao), *Nepeta tenuifolia* (Jing-Jie)	Standardized decoction packet / concentrated granule powder dissolved in warm water.	Antiviral & Immunomodulatory: Inhibits SARS-CoV-2 spike protein binding to ACE2 receptors, reduces viral replication, and suppresses pro-inflammatory cytokine storms ($IL-6, TNF-\alpha$).
Jing Si Herbal Tea<br><br>*(Jing Si Ben-Cao-Yin)*	*Artemisia argyi* (Ai-Ye), *Anisomeles indica* (Yu-Zhen-Cao), *Ophiopogon japonicus* (Mai-Dong), *Houttuynia cordata* (Yu-Xing-Cao), *Platycodon grandiflorus* (Jie-Geng), *Glycyrrhiza glabra* (Gan-Cao), *Perilla frutescens* (Zi-Su-Ye),	Commercial teabag infusion / ready-to-drink concentrated liquid packet.	Mucosal Protection & Anti-inflammation: Blocks viral entry at mucosal sites, demonstrates strong antioxidant activity, alleviates respiratory inflammation, and supports lung

Formulation Name	Botanical / Botanical-Mineral Compositions (Latin & Pin-Yin)	Primary Mode of Administration	Reported Mechanism & Preventive Profile
	Chrysanthemum morifolium (Ju-Hua)		function.
Qingwen Baidu Decoction *(Over-the-Counter Traditional Decoctions)*	*Gypsum fibrosum* (Shi-Gao), *Rehmannia glutinosa* (Sheng-Di-Huang), *Cornus officinalis*, *Scutellaria baicalensis*, *Coptis chinensis* (Huang-Lian), *Paeonia suffruticosa* (Mu-Dan-Pi)	Traditional raw herb decoction boiled at home / OTC concentrated extract granules.	Systemic Heat-Clearing & Detoxification: Traditional formula utilized for high fever, systemic inflammation, and severe viral infection risk reduction.

Source: Sourced and developed entirely by the authors based on primary survey data collected in this study ($N = 499$).
